# Supplementary material for: Antifibrotic Soluble Thy-1 Correlates with Renal Dysfunction in Chronic Kidney Disease
Source: Int J Mol Sci. 2023 Jan 18;24(3):1896. doi: 10.3390/ijms24031896 (PMC9916214; doi:10.3390/ijms24031896)
Supplement: Supplementary file 1 [file ijms-24-01896-s001.zip › ijms-2103233-supplementary.pdf]

Supplementary Materials

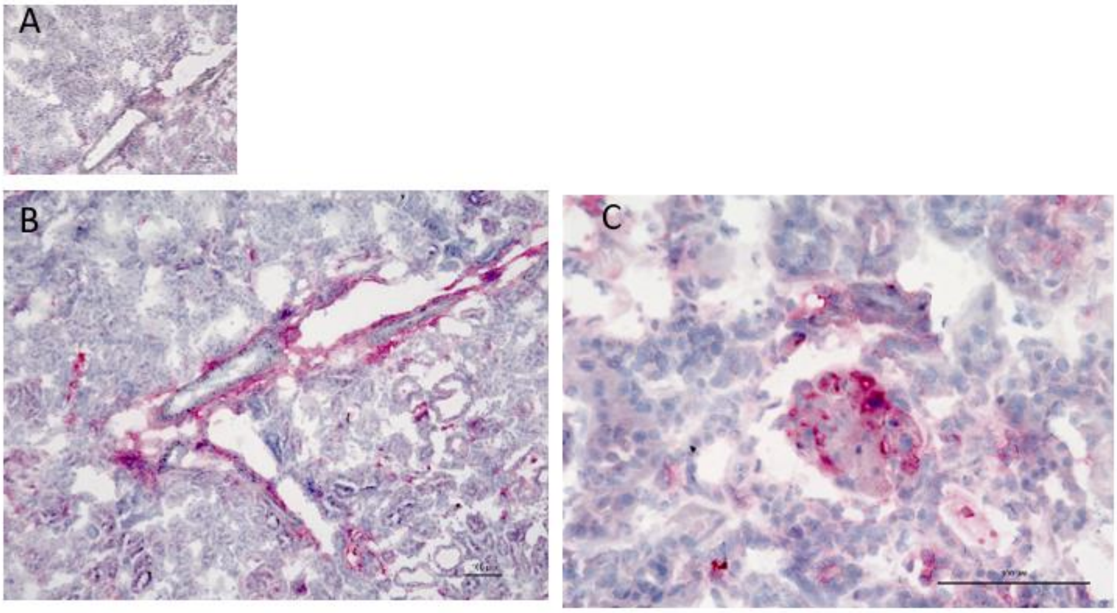

Figure S1. Thy-1 expression in a mouse model of chronic kidney disease (CKD). Thy-1 expression in kidney of CKD mouse model (eNOS-/- C57BLKS db/db) was detected by immunohistochemical staining with anti-Thy-1 antibody (red). Nuclei (blue) were counterstained with haematoxylin. Images represent one representative example of four different mice. A) Negative control with isotype control antibody. B/C Anti-Thy-1 antibody.

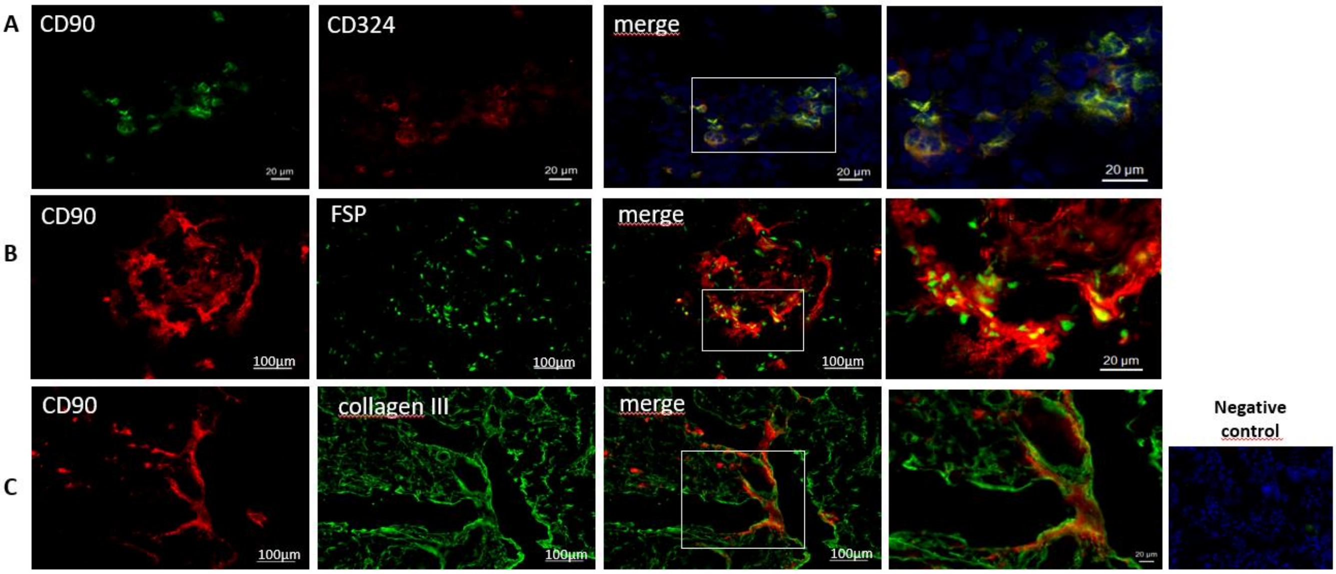

Figure S2. Thy-1 expression in a mouse model of chronic kidney disease (CKD). Expression of Thy-1, CD324 (A), collagen III (B) and Fibroblast-specific Protein (FSP; C) was detected by immunofluorescence stainingin kidney of CKD mouse model (eNOS-/- C57BLKS db/db). Images represent one representative example of three different mice. Isotype control antibodies were used as negative control. Nuclei were counterstained with DAPI (blue).

Table S1. Multivariate linear regression analysis of serum creatinine as a marker of renal function in cohort 1 (N = 120) with similar covariates compared to main analysis.

	**Multivariate linear regression analysis**	
	**β**	**p**
Sex	**-0.199**	**<0.001**
WHR	**-0.081**	**0.023**
Fasting glucose (mmol/l)	**0.165**	**<0.001**
HDL cholesterol (mmol/l)	**0.181**	**<0.001**
hsIL-6 (ng/l)	**0.365**	**<0.001**
sThy-1 (µg/l)	**1.291**	**<0.001**

Multivariate linear regression analysis of serum creatinine with anthropometric and biochemical markers, as well as sThy-1 in cohort 1 (N = 120). Multivariate regression analysis was calculated for serum creatinine (lg, dependent variable) adjusted for sex, WHR (lg), fasting glucose (lg), HDL cholesterol (lg), hsIL-6 (lg), as well as sThy-1 (lg). Non-normally distributed variables as assessed by Shapiro-Wilk-test were logarithmically transformed prior to multivariate testing (lg). Standardized β-coefficients and p-values are given. The coefficients corresponding to sex assume that males are coded such that females have a larger value. Abbreviations are indicated in Table 1.

Table S2. Multivariate linear regression analysis of serum creatinine as a marker of renal function in cohort 1 (N = 120) with well-established determinants of circulating creatinine in a large epidemiological analysis [19].

	**Multivariate linear regression analysis**	
	**β**	**p**
Age (years)	-0.045	0.169
Sex	**-0.140**	**<0.001**
WHR	-0.045	0.219
Smoking status	**0.116**	**<0.001**
Use of antihypertensive drugs	**-0.072**	**0.026**
Triglycerides (mmol/l)	**-0.240**	**<0.001**
sThy-1 (µg/l)	**1.007**	**<0.001**

Multivariate linear regression analysis of serum creatinine with anthropometric and biochemical markers, sThy-1, as well as well-established determinants of creatinine, in cohort 1 (N = 120). Multivariate regression analysis was calculated for serum creatinine (lg, dependent variable) adjusted for age (lg), sex, WHR (lg), smoking status, use of antihypertensive drugs, serum triglycerides (lg), as well as sThy-1 (lg). Non-normally distributed variables as assessed by Shapiro-Wilk-test were logarithmically transformed prior to multivariate testing (lg). Standardized β-coefficients and p-values are given. The coefficients corresponding to sex assume that males are coded such that females have a larger value. Abbreviations are indicated in Table 1.
